# The Conversion Rate of Laparoscopic Cholecystectomy to Open Cholecystectomy at King Abdulaziz Medical City, Jeddah, Saudi Arabia: Prevalence and Causes

**DOI:** 10.7759/cureus.63026

**Published:** 2024-06-24

**Authors:** Zaher Mikwar, Faisal F Aljadani, Abdulrahman K Alotaibi, Faris A Neazy, Nawaf H Alsaadi, Majed A Alzahrani, Abdullah Awadh

**Affiliations:** 1 Surgical Oncology, King Abdulaziz Medical City, Ministry of National Guard Health Affairs, Jeddah, SAU; 2 Medicine, King Abdullah International Medical Research Center, Jeddah, SAU; 3 Medicine, King Saud Bin Abdulaziz University for Health Sciences, Jeddah, SAU; 4 Infectious Disease, King Abdullah International Medical Research Center, Jeddah, SAU

**Keywords:** laparoscopic cholecystectomy, conversion to open cholecystectomy, laparoscopic day surgery, gall bladder diseases and gallstones, gallbladder

## Abstract

Introduction

Laparoscopic cholecystectomy has emerged as the preeminent surgical technique for cholecystectomy. However, in exceptional circumstances, surgeons may encounter significant obstacles that necessitate reverting to the traditional open approach, which has more undesirable complications. In this study, we aimed to identify the factors underlying conversion and to quantify its prevalence in the medical setting of King Abdulaziz Medical City (KAMC) in Jeddah to lower the rate of conversion.

Methodology

In this retrospective cross-sectional study, a non-probability consecutive sampling technique was utilized to include all patients over 16 years of age who underwent cholecystectomy at KAMC, Jeddah, between January 2009 and June 2022, excluding any patients with missing data.

Results

The total number of patients operated for cholecystectomy was 2,632, of which 1924 were female (73.1%) and 708 were males (26.9%). Of these patients, only 69 were converted to open (2.62%). Among them, 32 patients were in the age group >60, with the highest conversion rate (7.80%). The leading causes were adhesions in 55 cases (79.71%) and distorted anatomy in 31 cases (44.92%).

Conclusion

This study shows distorted anatomy and adhesions to be the leading causes of conversion from laparoscopic cholecystectomy to open approach at KAMC with a conversation rate of 2.62%. Furthermore, this article includes a higher number of patients in a longer period compared to other similar literature, which may give more accountable results that help reduce the conversion rate and complications.

## Introduction

Cholecystectomy is the surgical removal of the gallbladder due to many reasons. The two known surgical approaches are either open or laparoscopic cholecystectomy [1]. Laparoscopic cholecystectomy is considered the gold standard for the surgical treatment of gallbladder diseases [2,3]. The other surgical procedure is the open cholecystectomy, which is done through a right subcostal incision at the right upper abdomen where the right rectus abdominis muscle will be incised [4]. Open cholecystectomy was the standard approach for gallbladder removal in the past until laparoscopic cholecystectomy was invented more than 30 years ago and became the gold standard [5]. The laparoscopic approach became the superior technique because it has a shorter hospitalization period and more tolerable post-op pain and provides a faster recovery [6-9]. Other post-operative complications such as bleeding, infection, and possible incisional hernia may happen with both procedures but more commonly with the open technique [10]. However, there are certain situations where surgeons are forced to convert from laparoscopic to open cholecystectomy. Based on some studies, the causes were attributed to many factors such as male gender, patient’s age, acute cholecystitis, intra-peritoneal adhesions, inflammatory infiltration, distorted anatomy, bile duct injury, morbid obesity, previous abdominal surgery, presence of common bile duct stones, bleeding, gangrenous cholecystitis with extensive adhesions, limited surgical experience, and the lack of proper instruments [11-14].

The conversion rates of collected studies range from 2.5% to 7.78%, although some studies have shown a significantly higher rate. For example, in a study conducted at King Khalid University Hospital, Saudi Arabia, the conversion rate was as high as 23% [2]. In comparison, another study in the USA showed a conversion rate of 19.9% [5]. In contrast, in another study in Saudi Arabia, the conversion rate was as low as 0.9% [11].

Even though cholecystectomy is one of the most common procedures all over the world, there are not enough current studies to cover this topic in Saudi Arabia. Most of the research was conducted before 2010, which is considered old and may not represent the actual situation in addition to being either having a small sample size or covering only a short period [11-13].

In this study, we tried to identify the common indications of conversion in our institution to compare it with other national and international studies and add more helpful and accountable results that may help in enhancing the outcome of such a common procedure. The study's objectives focused on identifying the conversion rate from surgeries that started as laparoscopic cholecystectomy and then converted to open, identifying the main causes that led to this conversion, and associating the conversion rate with gender and age.

## Materials and methods

Study settings and design

The study was done as a retrospective cross-sectional study. The sampling technique used in this study was a non-probability consecutive sampling technique. It was conducted at King Abdulaziz Medical City, Jeddah, Saudi Arabia. These surgeries were performed by the general surgery department. The department comprises 14 general surgery consultants, two associate consultants, six assistant consultants, and 25 residents. Each surgeon performs approximately four to six surgeries per week, and about 60-70 surgical operations are performed by all surgeons per week. The number of laparoscopic cholecystectomies performed weekly ranges between seven and 10 surgeries. During the COVID-19 crisis, elective surgical procedures have drastically decreased, including laparoscopic cholecystectomy, but, recently, this number has been gradually returning to its normal ranges. The study was set between January 2009 to June 2022, and the number of patients was 2,632. The data were collected from the electronic Best-Care system, and the provided hard copies using the data collection sheet were reviewed by the supervising consultant.

Identification of study participants

The inclusion criteria were determined to be all patients aged 16 years and above who underwent cholecystectomy operation at King Abdulaziz Medical City in Jeddah from January 2009 till the end of June 2022, while the exclusion criteria were patients with missing data or patients younger than 16.

The data collection method was a chart abstraction completed by trained medical students. The data collection process started in July 2022 and continued till February 2023. The data used to complete the charts were obtained from hard copies and electronic files in the hospital’s electronic filing system named (Best-Care). A general surgeon consultant reviewed the data for accuracy.

Study measures

The patient data sheet included patients’ demographic information (age, weight, height, gender), comorbid illnesses, history (acute cholecystitis, biliary colic, episodes of acute cholecystitis, post-endoscopic retrograde cholangiopancreatography (ERCP) for biliary pancreatitis, previous abdominal surgeries, chronic cholecystitis), investigations such as ultrasound findings (thickened gallbladder wall, increased common bile duct diameter), leukocytosis, disturbed liver function test (alkaline phosphatase/total bilirubin), and outcomes (causes of conversion, histopathology). Data were entered into Microsoft Excel (Microsoft® Corp., Redmond, WA) to conduct data checking.

Statistical analysis

Frequency and percentages were used to describe categorical variables, and the chi-square test was used for comparison. The Statistical Product and Service Solutions (SPSS, version 26; IBM SPSS Statistics for Windows, Armonk, NY) was used to analyze the data in this study. A p-value of < 0.05 was considered statistically significant. Figure preparation was conducted using Microsoft Excel 2010.

No consent form was needed. This study used a data collection sheet for data collection. Participants’ privacy and confidentiality were assured, no identifiers were collected, and all the data were kept in a secure place within NGHA premises and accessed by the research team only.

## Results

Table 1 shows the total number of patients who underwent laparoscopic cholecystectomy, which was 2,632 (1,924 (73.1%) were females, and 708 (26.9%) were males). The age group 41-60 constituted the largest group in the study, with 1,061 (40.3%). The highest BMI group was 25-29.9 with 802 (30.5%) patients, while the BMI groups 25-29.9 and 30-34.9 combined constituted more than half the number of operated patients (56.6%). Out of 2,632 patients who attempted to undergo laparoscopic cholecystectomy, a total of 2,563 (97.38%) patients underwent laparoscopic cholecystectomy successfully, while 69 patients had to undergo conversion to open cholecystectomy. The laparoscopic to open conversion rate was 2.62%.

**Table 1:** Demographic Information of Patients of Laparoscopic Cholecystectomy (n=2632) *Indicates the highest percent.

Variables	Frequency (%)
Gender	
Male	708 (26.9)
Female	1924 (73.1) *
Age group	
16-25	212 (8.1)
26-40	949 (36.1)
41-60	1061 (40.3) *
> 60	410 (15.6)
BMI	
< 18.5	46 (1.7)
18.5-24.9	471 (17.9)
25-29.9	802 (30.5) *
30-34.9	687 (26.1)
35-39.9	398 (15.1)
> 40	228 (8.7)
Conversion rate	
Converted	69 (2.62)
Not converted	2563 (97.38) *

Most patients who converted to open cholecystectomy were females (56.52%). Among 708 patients, 30 males were converted (4.24%), while 39 from 1,924 female patients converted to the open procedure (2.03%) (OR: 2.138643, 95% CI: 1.31, 3.47). Fifty-six patients were older than 40, accounting for 81.16% of the total patients who were converted to open cholecystectomy (OR: 3.494863, 95% CI: 1.90, 6.42). Thirty patients from the 69 who converted to open cholecystectomy had a BMI of 30 and above (43.48%), while the BMI of the other 39 patients was less than 30 (56.52%) (OR: 0.766833, 95% CI: 0.47, 1.24) (Table 2).

**Table 2:** Converted Patients from Laparoscopic Cholecystectomy to Open Cholecystectomy Demographics (n=69) * Indicates the highest percent.

Variables	Frequency (%)
Gender	
Male	30 (43.5)
Female	39 (56.5) *
Age group	
16-25	2 (2.9)
26-40	11 (15.9)
41-60	24 (34.8)
> 60	32 (46.4) *
BMI	
< 18.5	3 (4.3)
18.5-24.9	17 (24.6)
25-29.9	19 (27.5) *
30-34.9	19 (27.5) *
35-39.9	7 (10.1)
> 40	4 (5.8)

The mean age for all the converted patients was 57.2 years, with a standard deviation of 16.69. The mean and standard deviation for male age was the highest (60.3 ± 15.48), while females recorded (54.82 ± 17.39). However, the male BMI mean was recorded at (26.91 ± 5.77), while the female BMI mean recorded the highest (30.79 ± 7.21) (Table 3).

**Table 3:** Distribution of Gender According to Age & BMI

Gender		Age	BMI
Male	Mean	60.30	26.91
	N	30	30
	Std. Deviation	15.48	5.77
Female	Mean	54.82	30.79
	N	39	39
	Std. Deviation	17.39	7.21
Total	Mean	57.20	29.10
	N	69	69
	Std. Deviation	16.69	6.85

Among the 69 patients who converted to open cholecystectomy, there were 25 patients diagnosed with acute cholecystitis (36.23%), 22 patients had chronic cholecystitis (31.88%), 15 patients were admitted with biliary colic (21.74%), six patients had Mirizzi syndrome (8.70%), and one patient had empyema of the gallbladder (1.45%) (Table 4).

**Table 4:** Post-operative Diagnosis in Converted Patients (n=69)

Post-operative diagnosis	Yes (%)	No (%)
Acute Cholecystitis	25 (36.2)	44 (63.8)
Chronic Cholecystitis	22 (31.9)	47 (68.1)
Biliary Colic	15 (21.7)	54 (78.3)
Mirizzi Syndrome	6 (8.7)	63 (91.3)
Empyema of the Gallbladder	1 (1.4)	68 (98.6)

Most abnormalities seen in ultrasound findings were gallbladder stones in 36 (52.17%), thickened gallbladder wall in 15 patients (21.74%), distended gallbladder in 14 patients (20.29%), increased diameter of the common bile duct in 13 patients (18.84%), common bile duct (CBD) stones in five patients (7.25%), and peri-cholecystic fluid in four patients (5.80%). In liver function tests, the most common abnormal findings were elevated total bilirubin in 13 patients (18.84%) and high alkaline phosphatase in 11 patients (15.94%), while leukocytosis was seen in 15 patients (21.74%). The most frequent comorbidities seen in patients who underwent open cholecystectomy were diabetes mellitus in 25 patients (36.23%), 24 with hypertension (34.78%), and 11 patients with dyslipidemia (15.94%). Sixteen (23.18%) of patients who converted had previous abdominal surgeries (Table 5).

**Table 5:** Clinical Findings in Patients Who Underwent Open Cholecystectomy (n=69)

Findings	Number of patients		%
Ultrasound			
Gallstones	36		52.17
Thickened gallbladder wall	15		21.74
Distended gallbladder	14		20.29
Increased diameter of CBD	13		18.84
CBD stones	5		7.25
Peri-cholecystic fluid	4		5.80
Liver Function Findings			
High total bilirubin	13		18.84
High alkaline phosphatase	11		15.94
WBC			
High	15		21.74
Comorbidities			
Diabetes mellitus	25		36.23
Hypertension	24		34.78
Dyslipidemia	11	15.94	
Previous abdominal surgeries	16	23.19	

There were many reasons for conversion, the two leading causes were adhesions in 55 cases (79.71%), and distorted anatomy in 31 cases (44.93%). The rest were Mirizzi syndrome in six patients (8.70%), CBD stones in two patients (2.90%), CBD injury in two patients (2.90%), and uncontrolled bleeding in one patient (1.45%) (Table 6).

**Table 6:** Reason for Conversion (n=69)

Reason of conversion	Yes (%)	No (%)
CBD stone	2 (2.9)	67 (97.1)
CBD injury	2 (2.9)	67 (97.1)
Bleeding	1 (1.4)	68 (98.6)
Mirizzi syndrome	6 (8.7)	63 (91.3)
distorted anatomy	31 (44.9)	38 (55.1)
Adhesions	55 (79.7)	14 (20.3)

Table 7 proves that there was a statistically significant relationship between conversion rate and age (p value = 0.000 < 0.05; X2 = 53.976), which indicates that conversion rate increases with age. The conversion rate among patients over 60 years old recorded the highest percentage of 7.8%. Other age groups, 16-25, 26-40, and 41-60, accounted for 0.9%, 1.2%, and 2.3%, respectively. Table 7 also proves that there was a statistically significant relationship between conversion rate and gender (p value = 0.002 < 0.05; X2 = 9.904), which indicates that the conversion rate increased with male gender, which recorded a higher percentage of conversion at 4.2%, while the conversion rate for female accounted 2.03%.

**Table 7:** Association Between Conversion Rate and (Age & Gender & BMI) (n=2632) * indicates a statistically significant relationship.

Demographics variables		Conversion rate		X^2^	p value
		Converted	Not converted		
Age	16-25	0.9%	99.1%	53.976	0.000*
	26-40	1.2%	98.8%		
	41-60	2.3%	97.7%		
	> 60	7.8%	92.2%		
Gender	Male	4.2%	95.8%	9.904	0.002*
	Female	2.0%	98.0%		
BMI	< 18.5	6.5%	93.5%	5.996	0.307
	18.5-24.9	3.6%	96.4%		
	25-29.9	2.4%	97.6%		
	30-34.9	2.6%	97.4%		
	35-39.9	2.0%	98.0%		
	> 40	1.8%	98.2%		

## Discussion

The study analysis concluded that the conversion rate from laparoscopic to open cholecystectomy was 2.62%. Adhesions and complex anatomy were found to be the most significant causes of conversion. Out of 69 patients converted to open cholecystectomy, 55 (79.7%) had adhesions, and 31 (44.93%) patients had distorted anatomy. Other factors, such as age and gender, correlated to the increased conversion rate. Men had a higher conversion rate, consisting of 4.24%, while the conversion rate of women reached 2.03%. Age and gender have been found to have a significant effect, leading to the conversion with P values of 0.000 and 0.002, respectively. A risk factor of having previous abdominal surgeries has been found in 16 (23.19%) conversions. Patients who had diabetes mellitus and underwent the conversion were 25 (36.23%), while 11 (15.94%) had elevated levels of alkaline phosphatase.

The study had comparable results in previously specified pre-operative risk factors for conversion such as male gender, patient’s age, intra-peritoneal adhesions, inflammatory infiltration, distorted anatomy, acute cholecystitis, bile duct injury, morbid obesity, previous abdominal surgery, and suspicion of common bile duct stones, diabetes mellitus, and elevated alkaline phosphatase. Older patients were more prone to undergo conversion to open cholecystectomy. In this study, the number of patients above 60 years was 410, and 32 of those had converted to open cholecystectomy, making up a conversion rate of 7.8%, constituting nearly half the number of patients who were converted to open cholecystectomy (46.38%). In contrast, in another study, it was found that the highest age group converted to open cholecystectomy is 65-year-old patients. Sixty-four patients out of 538 who were 65 (years and older) converted into open cholecystectomy, making up an 11.9% conversion rate. The male gender in this study had a conversion rate of 4.24%, while the female gender had a conversion rate of 2.03%. However, compared to another study, the male gender had a 9.6% conversion rate and a 3% conversion rate in the female gender [15]. In this study, distorted anatomy and adhesions were the leading factors that highly contributed to the conversion. Ghnnam et al. reported six (35.29%) distorted anatomy in converted patients, and Ashfaq et al. reported 37 (52.85%) adhesions in converted patients [5,16]. Comparing these results with this study gives remarkably similar results for these risk factors, as 31 (44.93%) patients had distorted anatomy and 55 (79.7%) patients had adhesions. The most common ultrasound finding was acute cholecystitis due to gallstones. In contrast, other less frequent findings were distended gallbladder, thickened gallbladder wall, and an increased common bile duct diameter. A study in Pakistan in 2019 showed that morbid obesity was a risk factor for conversion to open cholecystectomy [17]. However, this study's relationship between BMI and the conversion rate was insignificant. Coffin et al. reported a non-significant p value of 0.530 in the association between BMI and conversion. Similarly, this study showed a non-significant p value of 0.307 for any association between BMI and conversion from laparoscopic to open cholecystectomy [15]. Another study had identified it first to be a preoperative risk, but after comparing the results of 100 conversions with 100 randomly chosen laparoscopic cholecystectomy, it was found to be insignificant [14].

In several studies, the conversion rate varied from 2.5% to 7.78% [15-20]. Universally, a study conducted in the United States showed a conversion rate of 19.9% [5]. Conversely, a local study at King Khalid University Hospital, Saudi Arabia, showed a conversion rate of around 23% [2]. In contrast, another local study at King Fahad Hospital, Saudi Arabia, had a low percentage of 0.9% conversion rate [13]. Previous abdominal surgeries in this study were fewer than in other studies. Amin et al. had 40 (47.31%) patients who had a previous abdominal surgery compared to our 16 (23.19%), which is half as common in our converted patients than in the other study. Diabetes mellitus was in 25 (36.23%) of those who had conversions while Coffin et al. reported diabetes mellitus in about 15 (7.7%) patients who underwent the conversion. The same study also reported an elevated alkaline phosphatase level in 51 (60.71%) patients, while our study reported that 11 (15.94%) had elevated alkaline phosphatase levels [17].

Multiple studies determined many potential causes of conversion. The most common predictors were distorted anatomy, adhesions, male gender, older age, thickened gallbladder wall, morbid obesity, and diabetes mellitus. In this study, the conversion rate was 2.62%, and the most common reasons for conversion were male gender, older age, distorted anatomy, and adhesions.

Limitations and recommendations 

The length of the study had its benefits as well as its drawbacks. While trying to add as many operations as possible to solidify the results, this method had added limitations of its own, such as difficulty in providing hard copies and missing data, and patients less than 16 years of age were not included. The low number of converted patients can be considered as one of the limitations of this study. Finally, this single-hospital study limits the probability of generalizing the results. However, because this conversion is uncommon, the findings of this study solidify and add more helpful information to all previous literature. We recommend the upcoming studies to include all the age groups, study the association between BMI and the rate of conversion more intensively, and include the time consumed in the operation room before the conversion decision.

## Conclusions

The study included more patients through a period of 12 and a half years, possibly giving a more accountable result than other studies. Results of this study, including the low incidence rate of conversion and risk factors, found in converted patients such as male gender, old age, distorted anatomy, and adhesions continued to go alongside the results of most of the studies found in the literature. Distinguishing these risk factors may help recognize the possibility and need for conversion and may also help reduce the conversion rate. Other risk factors such as diabetes mellitus, morbid obesity, and hypertension were not entirely understood as to how they could affect the rate of conversion.
